# Ecological drivers of jaw morphological evolution in lepidosaurs

**DOI:** 10.1098/rspb.2024.2052

**Published:** 2024-12-11

**Authors:** Antonio Ballell, Hugo Dutel, Matteo Fabbri, Elizabeth Martin-Silverstone, Aleksandra Kersley, Chrissy L. Hammond, Anthony Herrel, Emily J. Rayfield

**Affiliations:** ^1^ Bristol Palaeobiology Group, School of Earth Sciences, University of Bristol, Life Sciences Building, 24 Tyndall Avenue, Bristol BS8 1TQ, UK; ^2^ Université de Bordeaux, CNRS, MCC, PACEA, UMR 5199, Pessac 33615, France; ^3^ Center for Functional Anatomy and Evolution, Johns Hopkins University School of Medicine, Baltimore, MD 21205, USA; ^4^ School of Physiology, Pharmacology & Neuroscience, University of Bristol, Biomedical Sciences Building, Bristol BS8 1TD, UK; ^5^ Mécanismes Adaptatifs et Evolution, UMR 7179, Muséum national d'Histoire naturelle CNRS, Paris 75005, France; ^6^ Department of Biology, Evolutionary Morphology of Vertebrates, Ghent University, Ghent 9000, Belgium; ^7^ Department of Biology, Functional Morphology Laboratory, University of Antwerp, Antwerp 2610, Belgium; ^8^ Naturhistorisches Museum Bern, Bern 3005, Switzerland

**Keywords:** Lepidosauria, ecomorphology, evolution, geometric morphometrics, evolutionary rates, disparity

## Abstract

Ecology is a key driver of morphological evolution during adaptive radiations, but alternative factors like phylogeny and allometry can have a strong influence on morphology. Lepidosaurs, the most diverse clade of tetrapods, including lizards and snakes, have evolved a remarkable variety of forms and adapted to disparate ecological niches, representing an ideal case study to understand drivers of morphological evolution. Here, we quantify morphological variation in the lower jaw using three-dimensional geometric morphometrics on a broad sample of 153 lepidosaur species. Our results suggest that phylogeny has significantly influenced mandibular shape evolution, and snakes have diverged from a lizard-like jaw morphology during their evolution. Allometry and ecological factors like diet, foraging mode and substrate also appear to drive the diversification of mandibular forms. Ecological groups differ in patterns of disparity, convergence and rates of evolution, indicating that divergent evolutionary mechanisms are responsible for the acquisition of different diets and habitats. Our analyses support that lepidosaurs ancestrally use their jaws to capture prey, contrary to the traditional view favouring lingual prehension as ancestral. Specialized or ecologically diverse lineages show high rates of jaw shape evolution, suggesting that morphological innovation in the mandible has contributed to the spectacular ecomorphological diversification of lepidosaurs.

## 1. Introduction

Phenotypic diversity in unequally distributed throughout the tree of life. During evolutionary radiations, clades explore different areas and extent of morphological space during the conquest of new ecological niches. Thus, ecological opportunity drives morphological diversification through the process of adaptation [1]. However, phylogenetic history and developmental processes may impose additional controls on the evolution of form [2–4]. The relative importance of each of these factors in the evolution of morphology, and the degree of interaction between them, is a major question in evolutionary biology, and appears to vary depending on the biological system and taxonomical group of study (e.g. [5–7]). Thus, identifying the evolutionary patterns and drivers of morphological diversification within and across lineages is fundamental to understand how clades radiate and biodiversity is generated.

Lepidosaurs are the clade of diapsids comprising lizards, snakes and the tuatara, and with over 11 000 species, represent the most speciose group of tetrapods today [8]. Since their origin at more than 240 Ma [9], lepidosaurs have diversified into a myriad of sizes and body plans, expressed in a remarkable disparity of cranial and postcranial morphologies. Among living species, the range in body size spans three orders of magnitude, as exemplified by the approximately 17 mm long *Sphaerodactylus* geckos and the approximately 10 m long green anaconda [10]. Extremes in large body size become even more dramatic when extinct mosasaurs are considered (up to 17 m in length; [11]). Disparity in body form is reflected in the different degrees of body elongation, and reduction or modification of limb elements seen in multiple lineages, with snake-like body plans evolving at least 25 independent times [12]. Similarly, lepidosaurs show a rich variety in skull configurations [13,14] shaped by the loss and gain of skull bones during their evolutionary history [15,16], and the acquisition of different kinds and degrees of cranial kinesis [17]. As a result of this diversification of forms, lepidosaurs have conquered diverse ecological niches across most of the globe [18,19]. In the terrestrial realm, lepidosaurs inhabit diverse microhabitats on the ground, in trees, on rocks and in crevices [20,21], while several lineages of lizards and snakes have independently evolved fossorial lifestyles, usually associated with limb reduction and body elongation [22]. Moreover, various groups have independently acquired semiaquatic habits [23], while a few snake lineages and the extinct mosasaurs adapted to aquatic environments [11,24]. Diet is another aspect of ecology that has played a major role in the diversification of lepidosaurs [18,25,26]. Living species show an outstanding breadth of feeding habits, comprising predators feeding on vertebrate (carnivores) and invertebrate prey (insectivores), and species that feed partly (omnivores) or strictly (herbivores) on plant matter [27,28]. This striking diversity of forms and ecologies makes lepidosaurs an exceptional model system to understand the drivers of morphological evolution and biodiversity.

The skull is a particularly interesting system to decipher the ecological drivers of morphological evolution, since it is involved in multiple functions like feeding, locomotion and defence [29]. In lepidosaurs, the relationship between skull morphology and ecology has been investigated, revealing that both ecological aspects such as diet or habitat, and other factors like phylogeny, allometry and heterochrony, have shaped the evolution of this structure [7,30–34]. Surprisingly, the mandible has received much less attention than the cranium. Despite being historically assumed to be more tightly linked to feeding [35], a two-dimensional morphometric study of lower jaw shape in lizards suggested that its relationship with diet is weak [31]. This may be because the tongue has a central role in food acquisition and processing, as lepidosaurs are the clade of amniotes that shows the greatest diversity in tongue morphology and function [36,37]. Prey capture is achieved with either the tongue, the jaws or both, and the type of prehension used varies among clades. Iguanian lizards use lingual (i.e. tongue) prehension, while most other squamates, including snakes, capture prey with their jaws [18,36–38]. *Sphenodon* primarily uses lingual prehension, but it catches large prey with the jaws [37,39]. This led to the interpretation of lingual prehension as the ancestral condition of lepidosaurs and squamates [18]. However, this hypothesis has more recently been undermined by the rejection of an early diverging position for Iguania in all recent phylogenies of Squamata [40–42], and the discovery of species that use both types of prehension within clades that were considered exclusively jaw feeders [43–45], rendering the evolutionary pattern of prehension in lepidosaurs unclear. Moreover, the degree to which prehension mechanism and other aspects of ecology such as diet or habitat are related to the morphological evolution of the lower jaw remains elusive.

In this study, we present the first broadscale quantification of mandibular shape variation across all major clades of living lepidosaurs (153 extant species), and one of the very few studies of this kind in a major vertebrate clade [46,47]. For the first time to ourknowledge, this study quantifies the significance of multiple potential drivers of morphological evolution (prehension, foraging mode, substrate, dietary ecology, phylogeny and allometry) using three-dimensional geometric morphometrics and phylogenetic comparative methods. We test the hypothesis that diet and ecology are the main drivers of jaw shape evolution, and explore patterns of disparity, convergence and evolutionary rates of jaw morphology among ecological groups. We also estimate the tempo of mandibular evolution in Lepidosauria, predicting that high evolutionary rates are related to ecological innovation. Finally, we reconstruct the evolution of prehension mechanisms to test the hypothesis that prey capture was ancestrally performed via lingual prehension in lepidosaurs.

## 2. Material and methods

### (a) Geometric morphometrics

Mandibular shape was captured using a landmark-based three-dimensional geometric morphometrics approach. Mandible three-dimensional models were generated from computed tomograph datasets (electronic supplementary material, table S1). A total of 125 landmarks were digitized on each model in Stratovan Checkpoint (Stratovan), comprising 10 fixed landmarks and 11 semi-landmark curves (electronic supplementary material, figure S5 and table S2). Landmarks were digitized on the left hemi-mandible, since analysing shape in one side of bilaterally symmetrical structures is appropriate to capture morphological variation at interspecific and macroevolutionary levels [48].

Curve semi-landmarks were first resampled along each curve to ensure equal spacing using the functions from [49] in R. Semi-landmarks were slid against the Procrustes consensus minimizing bending energy in three steps to ensure spatial homology [50] with the slider3d function of the Morpho package [51]. Slid landmark coordinates were superimposed during generalized Procrustes alignment using the gpagen function of the geomorph package [52], removing variation due to position, rotation and scaling. Finally, the Procrustes coordinates were ordinated in a principal components analysis (PCA) with the gm.prcomp function. In addition to standard PCA, we also performed phylogenetic PCA on the Procrustes coordinates in order to incorporate phylogeny during ordination and minimize its effect [53].

### (b) Multivariate statistics

The strength of the phylogenetic signal (see the electronic supplementary material for phylogeny information) in the shape data was tested by computing the *K*_mult_ statistic [54] with the physignal function of geomorph. Similarly, we computed the *K*_mult_ for the log-centroid size values to measure phylogenetic signal in the size proxy with the phylosig function in phytools [55]. We also tested for allometric signal in shape data using standard and phylogenetic Procrustes analysis of variances (ANOVAs) with log-centroid size using the procD.lm and procD.pgls functions in geomorph [52]. We also tested whether lizards and snakes have different allometric trajectories by including these groups in a phylogenetic regression of allometry. To correct for the effect of size on shape, we extracted allometry-free shape coordinates from the residuals of the phylogenetic generalized least squares (PGLS) regression with the detrend_shapes and expected_shapes functions of the morphospace R package [56]. These allometry-corrected shapes were subjected to PCA and phylogenetic PCA with the gm.prcomp function [52].

We tested whether ecological variables (diet, prehension mechanism, foraging mode and substrate; electronic supplementary material) have a significant effect on jaw shape variation by applying phylogenetic ANOVAs in geomorph [52] on both the original Procrustes coordinates and the allometry-free phylogenetic principal components (pPCs) accounting for 95% of shape variation (first 28 pPCs). To visualize morphological differences in jaw shape among dietary categories and foraging modes, we reconstructed mean theoretical jaw shapes from the allometry-corrected Procrustes coordinates (electronic supplementary material, text and figure S1).

Convergence in jaw shape within ecological categories was tested in the RRphylo package [57]. We used the search.conv function, based on phylogenetic ridge regression, on the allometry-free PCs accounting for 95% of the variation (first 15 PCs) to compute convergence metrics (mean theta angle of morphological vectors and its associated *p*‐value) for each ecological group [58]. Morphological disparity of the mandible was calculated between clades, dietary, prehension, foraging and substrate groups using the dispRity.per.group function in the dispRity package [59] with sum of variances as metric. In the case of the test of disparity per clade, *Sphenodon* was excluded as it is the single member of Rhynchocephalia. The tests were run on both the original and corrected Procrustes coordinates, and the significance of pairwise disparity comparisons was assessed with Wilcoxon tests with Bonferroni correction for multiple comparisons using the test.dispRity function (electronic supplementary material, tables S7–S11).

We reconstructed rates of evolution of jaw shape per dietary, prehension and foraging group. We used a sample of 100 trees from [42] to accommodate for phylogenetic uncertainty, which were pruned and grafted to include *Sphenodon* (electronic supplementary material). With this sample of 100 posterior trees, we fitted a series of Markov (Mk) models to the diet, prehension, foraging and substrate data differing in the transition rate model (equal rates, symmetrical rates and all rates different) with the fit_mk function of the castor package [60] and selected the best one per ecological category based on their Akaike information criteria and log-likelihood. Then, each selected model was used to perform a stochastic character mapping of the ecological data on the tree sample with the make.simmap function of phytools [55]. Multivariate linear models were fitted to the Procrustes coordinates (both original and corrected) and character-mapped phylogenies using a multi-state Brownian motion (BMM) model and penalized-likelihood method within the mvgls function of the mvMORPH package [61]. Model parameters were extracted for the 100 iterations of each analysis and summarized as the rates of jaw morphological evolution per ecological category, as in previous studies [46,47]. Analyses were repeated on a sub-sample consisting of the tuatara and lizards to explore jaw shape variation and its drivers in these groups in more detail. Although this grouping is artificial and paraphyletic, these analyses serve as a test of the effect of the specialized snake jaw morphology with unfused symphysis and an intramandibular joint on the patterns recovered for all lepidosaurs (see electronic supplementary material, table S5 and figures S2–S4).

Ancestral state estimation approaches were applied to reconstruct the evolution of prehension mechanisms in Lepidosauria using phytools [55] and four different transition state models. Prehension ancestral state probabilities were estimated at internal nodes by averaging the results with leave-one-out cross-validation (electronic supplementary material) in treesurgeon (https://github.com/evo-palaeo/treesurgeon).

Evolutionary rates of jaw morphology were reconstructed in BayesTraits v4 (http://www.evolution.reading.ac.uk) using the allometry-free pPC scores representing 95% of shape variation (28 pPCs). In particular, we applied the Variable Rates model, which uses a reversible-jump Markov chain Monte Carlo algorithm to identify significant rate variation across the tree. We ran the analysis with five independent chains, each one with 200 000 000 iterations, sampling each 20 000 iterations and discarding a burn-in of 30 000 000. In order to estimate the marginal likelihoods, we used a stepping stone sampler, with 200 stones sampled for 10 000 iterations. Convergence of chains was assessed by examining the trace plots with the BRprocessR R package (https://rdrr.io/github/hferg/BTprocessR/), and estimating the effective sample size and running a Gelman & Rubin’s diagnostic convergence test [62] with the effectiveSize and gelman.diag functions in the CODA package [63]. The marginal likelihoods of the Variable Rates results were compared with the output of a single-rate Brownian motion model using the Bayes factor, revealing significantly more support for the Variable Rates model (BF < 6 725). The outputs of the Variable Rates analysis were summarized using the rjpp function of the BTRTools package (https://github.com/hferg/btrtools), and the average evolutionary rates were plotted onto the phylogeny using plotBranchbyTrait in phytools.

## 3. Results

### (a) Mandible shape variation

**Figure 1.**
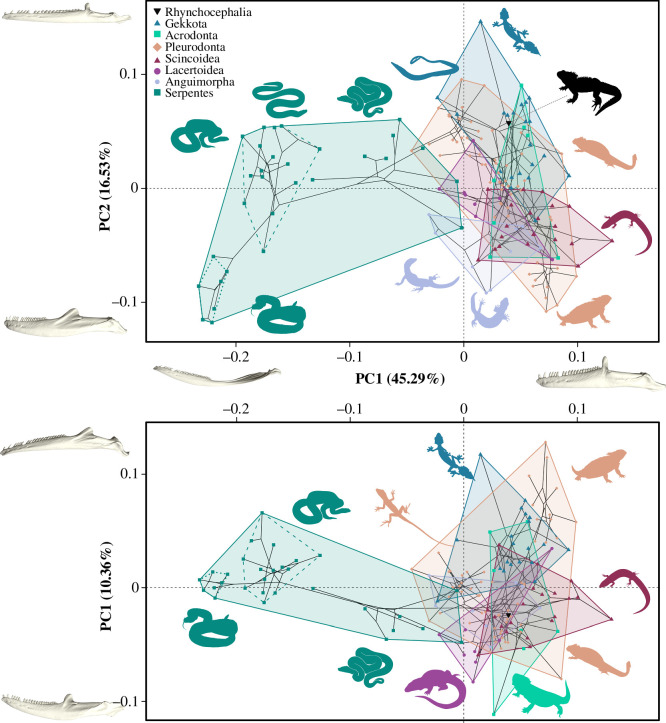
Phylomorphospace of lower jaw shape in Lepidosauria. Top panel shows the PC1–PC2 phylomorphospace; bottom panel shows the PC1–PC3 phylomorphospace. Extreme jaw morphologies are represented at the positive and negative ends of the three principal coordinate (PC) axes. Convex hulls indicate different lepidosaur clades. Within Serpentes, dashed hulls represent Colubroidea, and dotted hulls represent Viperidae. Silhouettes from PhyloPic.org.

Lower jaw shape variation is represented in the morphospace resulting from the PCA, with its first three PC axes summarizing over 72% of shape variance (figure 1). PC1 (approx. 45% of variation) expresses differences in jaw curvature, robustness and dimensions of the coronoid and retroarticular processes, essentially representing the transition between lizard-like and snake-like jaw morphologies. The second PC axis (approx. 17% of variation) separates jaws based on the length of the toothrow, width of the coronoid process, degree of ventral curvature of the jaw ramus, direction and width of the retroarticular process and size of the angular process. Finally, PC3 (approx. 10% of variation) expresses differences in the mediolateral and dorsoventral curvature of the mandible, the posterior deflection of the coronoid process and the orientation of the jaw joint and the retroarticular process. Interestingly, while PC1 expresses the highest proportion of variance for the whole sample, the major axis of variance for non-ophidian lepidosaurs is PC2 (see electronic supplementary material, Results and figure S2).

Lizards and snakes occupy different areas of morphospace with minimal overlap, falling on the positive and negative sides of PC1, respectively (figure 1). Gekkotans, with straight, gracile jaws, long tooth rows and slender coronoid processes, occupy the positive quadrants of PC2 and PC3. Scincoids are mainly restricted to the negative sides of PC2 and PC3 owing to their robust, ventrally curved jaws with large processes. Lacertoids fall at the centre of the PC1–PC2 morphospace and on the negative side of PC3, represented by ventrally convex, mediolaterally straight jaws with prominent retroarticular processes. Anguimorphs partly overlap in morphospace with scincoids, although varanids move towards the snake area on negative PC1. Iguanians occupy a wide range of morphospace along PC2 and PC3, with Pleurodonta spanning the largest area. Within this group, liolaemids are the closest to the centre of the morphospace, anoles plot on the positive side of PC2 with their straight, slender jaws with long tooth rows, and phrynosomatids diverge towards extreme negative PC2 and positive PC3 representing strongly medially bowed jaws with short tooth rows. Acrodontans also span both sides of PC2 and PC3, with the latter axis separating chameleons on the positive side (straight ventral margins, medially curved jaw and short retroarticular process) and agamids on the negative side. Snakes are restricted to the negative side of PC1, and span a wide range of PC2 but only central positions of PC3. ‘Henophidian’ snakes (e.g. pythons, boas and other lineages) are the closest to lizards in morphospace, with some fossorial and aquatic species (*Cylindrophis*, *Calabaria* and *Achrochordus*) overlapping with them. From their last common ancestor, caenophidian snakes diverge in two directions: Colubroidea move towards positive PC2 with their straighter jaws and longer tooth rows, while viperids evolve into extreme negative areas of PC1 and PC2, with their strongly curved jaws with very short tooth rows. Finally, *Sphenodon* overlaps with iguanians, closest to iguanids.

### (b) Phylogenetic and allometric signals

**Figure 2.**
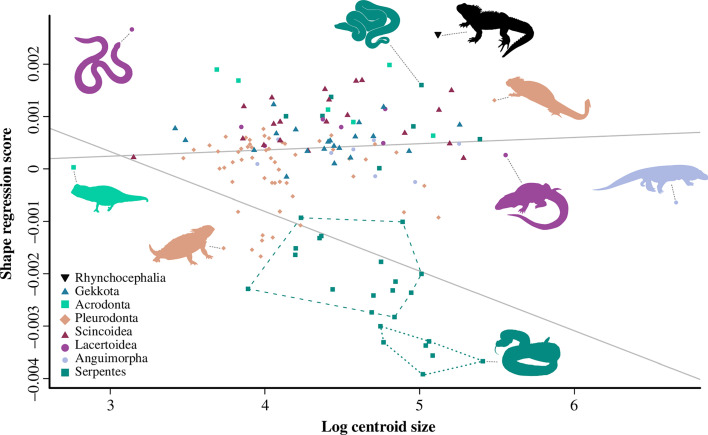
Phylogenetic regression of allometry of jaw shape on size. Regression lines represent allometric trends in lizards and snakes. Within Serpentes, dashed hulls represent Colubroidea, and dotted hulls represent Viperidae. Silhouettes from PhyloPic.org.

Phylogeny has a significant effect on both jaw shape (*K*_mult_ = 0.57, *p* < 0.001) and centroid size (*K*_mult_ = 0.30, *p* < 0.001). Similarly, linear and phylogenetic regressions of size (log centroid size) on jaw shape reveal that allometry is also significant but weak (*R* ^2^ = 0.056, *p* < 0.001; *R* ^2^ = 0.016, *p* < 0.01; electronic supplementary material, table S3). A phylogenetic regression with grouping shows that lizards and snakes have different allometric trajectories (figure 2), as suggested by the significant interaction between centroid size and group (*R* ^2^ = 0.019, *p* = 0.008). Allometry is also significant but weakly correlated with jaw shape in the lizard-only dataset (*R* ^2^ = 0.023, *p* = 0.005).

### (c) Influence of ecology on jaw shape

Correlation between jaw shape and four ecological variables (diet, prehension, foraging mode and substrate) was tested with phylogenetic ANOVAs. At the alpha level of 5%, none of the four ecological variables tested had a significant effect on uncorrected jaw shape variation (electronic supplementary material, table S4). After phylogenetic and allometric correction, diet, foraging mode and substrate become significant (electronic supplementary material, table S4). The morphological variation associated with differences in diet is visualized in the theoretical mean jaw shapes per dietary group (electronic supplementary material, figure S1), with carnivores and herbivores showing the starkest differences. The carnivore mean jaw shape is slender and curved and has a low coronoid process and narrow retroarticular process. In contrast, the herbivore morphology is characterized by a deep, robust jaw with a tall and wide coronoid process and well developed angular and retroarticular processes. The omnivore shape is intermediate between the two, while the insectivorous jaw shape is curved mediolaterally, and has a straight ventral margin and a ventrally deflected retroarticular process.

### (d) Mandibular disparity per clade and ecological group

**Figure 3.**
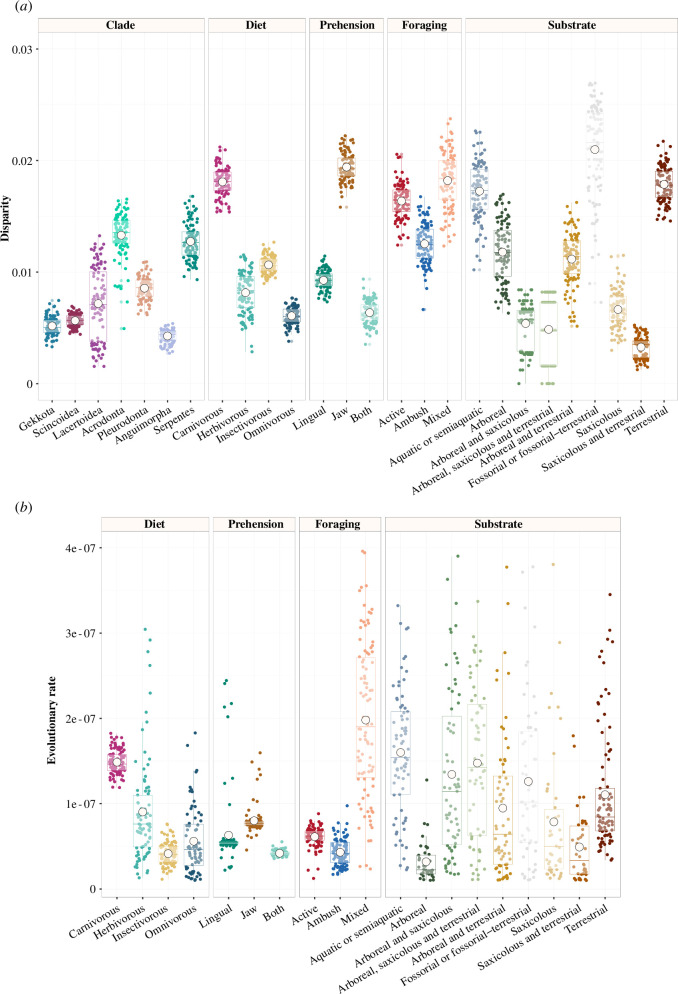
Morphological disparity and rates of evolution of the lower jaw per clade and ecological category. (*a*) Disparity per clade and ecological group as sum of variances. (*b*) Evolutionary rates per ecological group. Boxes represent the median with confidence intervals, white circles represent the mean.

Morphological disparity of the mandible varies among clades and ecological groups (figure 3; electronic supplementary material, tables S7–S11). Among clades, Acrodonta shows the highest disparity, followed by Serpentes and Pleurodonta, and Anguimorpha is the least disparate clade (figure 3*a*). Carnivores are remarkably more disparate in jaw shape than the other dietary groups, followed by insectivores, and with omnivores least so. Jaw prehension is the prey capture mode associated with the highest disparity in lepidosaurs (figure 3*a*). Mixed foragers show the highest disparity and ambush foragers the lowest (figure 3*a*). Of the substrate-use groups, fossorial/fossorial–terrestrial taxa show the highest jaw disparity, followed by terrestrial and aquatic–semiaquatic species (figure 3*a*).

### (e) Evolutionary rates and ecology

Rates of jaw shape evolution vary across ecological groups. Carnivores show the highest rates among dietary categories, followed by herbivores, and insectivores are the slowest evolving (figure 3*b*). Jaw prehension leads to the fastest rates among prehension mechanisms (figure 3*b*). Mixed foragers show the highest rates, followed by active foragers (figure 3*b*). Aquatic–semiaquatic taxa show the fastest rates among substrate-use group, followed by arboreal–saxicolous, arboreal–saxicolous–terrestrial and fossorial/fossorial–terrestrial species (figure 3*b*).

### (f) Convergence in jaw shape

Convergence in lower jaw morphology, as measured by the angle of phenotypic vectors theta (*θ*), was significant within several ecological groups (electronic supplementary material, table S6). Convergence was significant in all dietary groups except for carnivores, being particularly strong in herbivores (*θ* = 69°) and omnivores (*θ* = 72.7°). Among prehension modes, only taxa that use both types of prehension show significant convergence (*θ* = 58.1°), and for foraging groups, convergence is significant in ambush foragers (*θ* = 84.1°). Finally, three substrate categories show significant convergence: arboreal (*θ* = 79.3°), arboreal–saxicolous (*θ* = 55.7°) and saxicolous–terrestrial (*θ* = 64.2°).

### (g) Rates of evolution of jaw morphology

**Figure 4.**
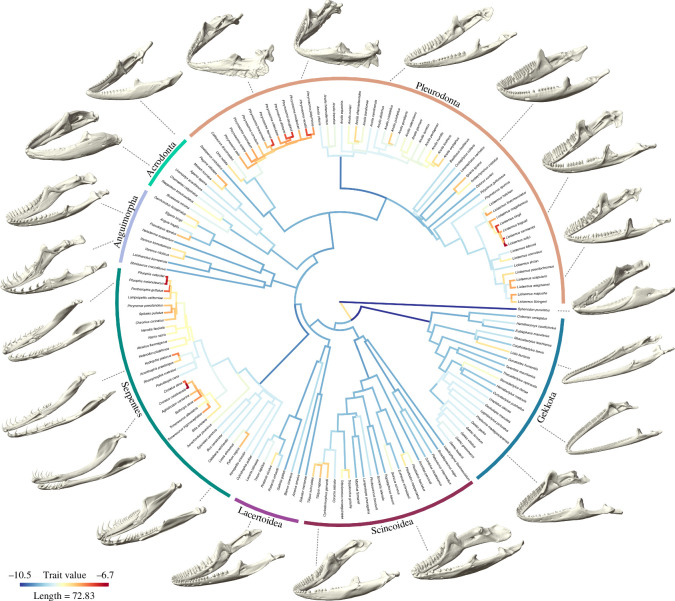
Rates of evolution of mandibular morphology in Lepidosauria. Branch-specific evolutionary rates (log-transformed) represented by a colour gradient, where red represents high rates and blue, low rates.

Branch-specific evolutionary rates of mandibular morphology reveal fast rates at the origin of Squamata, as well as in several lineages (figure 4). Gekkota shows very slow rates at its base, with generalized low rates across all its branches except for the one leading to the pygopodid *Lialis*. Similarly, low rates of evolution are pervasive within Scincoidea, Lacertoidea and Anguimorpha. Within Serpentes, all ‘henophidian’ branches show slow rates of evolution except for *Python*, but accelerated evolutionary rates are independently seen in caenophidian lineages like viperids (the genus *Crotalus* being the fastest evolving), the sea snake *Hydrophis*, and colubrids, especially in the genus *Pituophis*. The internal branches of Acrodonta show moderate evolutionary rates of jaw morphology, with accelerated rates seen in the agamids *Pogona* and *Moloch*. Among Pleurodonta, high evolutionary rates are present in all branches of horned lizards (genus *Phrynosoma*). Deceleration in rates of jaw evolution occurs at the base of Dactyloidae, although moderate rates are present in its descendant branches. Iguanids (*Iguana* and *Amblyrhynchus*) show moderate to high evolutionary rates, and fast rates also occur on different branches within *Liolaemus*.

### (h) Evolution of prehension mechanisms

**Figure 5.**
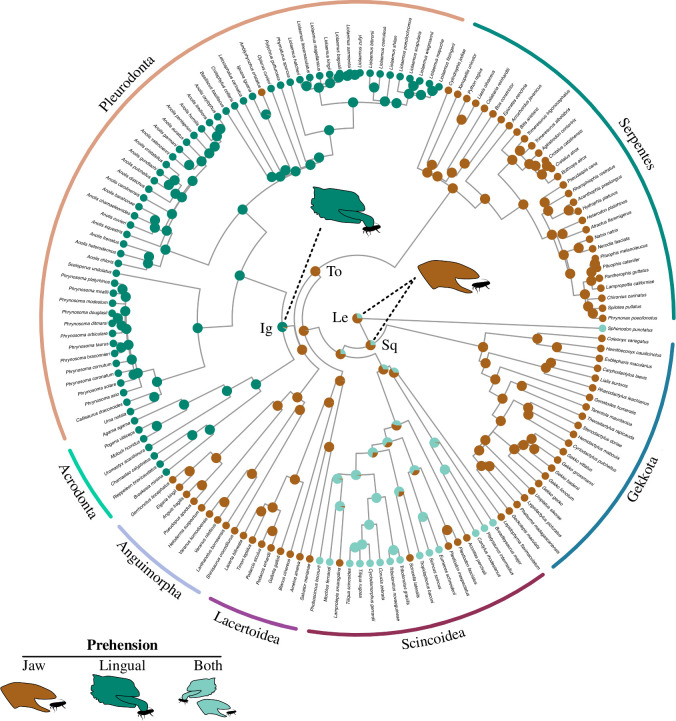
Ancestral state reconstruction of prehension mechanisms. Pie charts represent the probability of each character state at each node, as the consensus of a leave-one-out cross-validation on four different models of character state transition. Abbreviations: Ig, Iguania; Le, Lepidosauria; Sq, Squamata; To, Toxicofera. Prehension mode icons modified from [18].

The ancestral state reconstruction of prehension mechanism supports that the ancestral condition in Lepidosauria is either jaw prehension (72% probability) or both types of prehension (28%; figure 5). Similar estimations are recovered for Squamata and Unidentata (74% jaw prehension, 26% both), while the ancestral condition in Gekkota is unambiguously jaw prehension (>99%). Jaw prehension is the most likely ancestral state for all Scincoidea (67 and 33% for both modes), and almost equally likely to both types of prehension in Scincidae (55 and 45%). Episquamata and most of its major clades unambiguously have jaw prehension as their ancestral condition. The only major lineage with ancestral lingual prehension, with a probability of more than 90%, is Iguania.

## 4. Discussion

The evolution of morphological attributes is often explained in the light of adaptation to the environment [64], although the relationship between morphology, functional performance and ecology is complex and not always straightforward [65]. In this study, we quantitatively characterized morphological variation of the mandible in all lineages of extant lepidosaurs and tested the importance of phylogeny, allometry and ecology in driving the evolution of this structure. Our results show that phylogenetic history has influenced the evolution of the lower jaw, echoing previous studies that found significant phylogenetic signal in the morphology of the cranium among squamates [30,32,33]. The main axis of morphological variation represents the transition from a lizard-like to a snake-like mandible, evidencing the specialized jaw morphology in Serpentes. Phylogenetic signal in mandible shape is strong even within lizards, as reflected in a two-dimensional morphometric study [31], and suggests that phylogenetic heritage has acted as a primary force structuring morphological variation of the jaw in lepidosaurs. As such, clades of squamates differ in jaw disparity and occupy different areas of morphospace. Among lizards, iguanians prove to be the clade with the greatest jaw disparity, with pleurodontans occupying the largest area of morphospace, evidencing their wide range of morphologies, which include gracile jaws of *Anolis*, the robust mandibles of iguanids and the short and arched jaws of horned lizards (*Phrynosoma*). However, acrodontans, the clade including chameleons and agamids, have the highest jaw disparity among all lepidosaurs. Agamids have been previously shown to have remarkable disparity compared with other iguanian groups as a result of their radiation into very diverging morphologies [66]. This interpretation agrees with our results for the mandible, as some agamids (*Moloch*, *Pogona*) occupy extreme positions in PC2 and PC3. This suggests that acrodontans have diversified into distant and isolated regions of morphospace compared with other clades. In contrast, the other lizards (Gekkota, Scincoidea, Lacertoidea and Anguimorpha) show lower jaw disparity but reduced overlap in morphospace, indicating an early split in jaw evolution across all these groups.

Our morphometric analyses emphasize the morphological uniqueness of the snake mandible among lepidosaurs. Snakes occupy the largest area of morphospace, mostly non-overlapping with that of other lepidosaurs, and show the second highest disparity among all clades. The phylomorphospace shows the progressive modification of the jaw throughout snake evolution, with ‘henophidian’ snakes showing a more conservative morphology represented by a tall coronoid process, long tooth rows and straight jaws. Despite their lower degree of jaw modification, these early-diverging lineages exhibit derived feeding traits, such as the increased gapes seen in boids and pythonids [67,68]. Interestingly, some non-caenophidian snakes with fossorial (*Cylindrophis* and the boid *Calabaria*) and aquatic habits (*Acrochordus*) have evolved towards the area of morphospace occupied by lizards, suggesting a possible link between these ecologies and their morphological conservativeness. Their ‘lizard-like’ jaw shapes might be also a reflection of their feeding ecology, for instance, the robust jaws of *Cylindrophis* are likely related to powerful restraining bites used by these snakes [69]. In contrast, colubroid snakes clearly depart from lizards in jaw shape space, consistent with the identification of an exceptional phenotypic and dietary diversification that significantly expanded trophic niches in this clade [42]. One particular lineage, the viperids, pushes the limits of mandible shape innovation with their extremely curved jaws with short tooth rows, a unique shape that is likely related to the ingestion of bulky prey such as rodents [67].

Besides phylogeny, our findings show that other factors have driven jaw shape evolution in lepidosaurs, one of them being allometry. Evolutionary allometry of the skull has been investigated in individual clades of squamates like agamids [70], lacertids [71] and snakes [72] but not so frequently in the whole clade [32,33]. Despite its significance, it is shown here that allometric effects are relatively weak as they explain a small proportion of jaw shape variation, similar to the pattern observed in crania [33]. Most importantly, allometric regressions of shape show that snakes have a different allometric slope compared with the rest of Lepidosauria, with an idiosyncratic pattern of morphological change as they increase in size. A divergent evolutionary allometry pattern has also been identified in the snake cranium, thought to be associated with the evolution of fossoriality at the origin of this clade [32]. For the mandible, the distinct allometric patterns might reflect unique size-dependent functional demands linked to the specialized feeding mode of snakes, and the relative size of their prey [67]. While lizards use their jaws to immobilize and process prey items, snakes have evolved alternative ways to subjugate their prey, like constriction and venom, and swallow prey whole [73], releasing the mandible from substantial stress from biting on large animals.

Ecological variables have varying effects on mandibular shape variation in Lepidosauria, mostly underlying the stronger phylogenetic signal. Diet is a fundamental factor of ecology that has shaped the evolution of the skull of different groups of vertebrates [74,75], including lepidosaurs [33], although the relationship between skull shape and dietary ecology is not always tight [76,77]. We found that the signal of diet on jaw morphology emerges after phylogenetic correction of shape data, and that mandibular morphotypes associated with dietary categories differ in certain characters such as jaw robustness and the size of processes. For instance, on average herbivores have deeper mandibles with taller coronoid processes and wider retroarticular processes, a morphology consistent with their deeper skulls [78] and higher bite forces [79,80], which, in combination with more complex, cusped teeth [81], are adaptations to processing tougher, fibrous plant matter. In contrast, carnivores possess more elongate, gracile jaws, a trait that is thought to favour speed to catch prey [78]. Besides morphological differences, we find dietary groups show different patterns of disparity, convergence and evolutionary rates. Herbivores exhibit low disparity in jaw shape, a feature that is likely associated with their strong morphological convergence, countering a previous idea that herbivorous lizards did not converge in similar jaw shapes but in mechanical advantage [31]. Since those results were based on a two-dimensional morphometric analysis, capturing shape in three dimensions, as we do here, might be important to tease apart morphological patterns of convergence. Despite their limited disparity, herbivores show high evolutionary rates of jaw shape, suggesting that the evolution of herbivory in lizards was achieved by means of convergent exploration of similar mandibular shapes through rapid evolutionary changes. On the other hand, insectivores show the highest disparity among lizards, and although convergence in jaw shape is significant, it is not strong enough to prevent the evolution of diverse jaw morphologies, ranging from the short and wide jaws of phrynosomatids to the slender jaws of insectivorous geckoes, to feed on the diverse range of arthropod prey. Thus, our results suggest that diet has impacted the evolution of jaw shape in lepidosaurs through the incorporation of plants and invertebrates.

We found a strong association between jaw shape and substrate in line with previous work that identified habitat as an important driver of skull evolution in lepidosaurs [33]. In particular, we found the highest jaw disparity and rates of evolution are associated with fossorial, aquatic and terrestrial–arboreal habits. Moving and feeding in subterranean and aquatic environments impose specific demands on body shape, driving the evolution of head shape in different groups of squamates [82–84]. We find these habitats also catalyse the evolution of mandibular morphology, suggesting that ecomorphological innovation in the lower jaw is associated with the exploitation of diverse ecological niches by fossorial, aquatic and semi-aquatic squamates.

Prey prehension has been considered a fundamental aspect of lepidosaur feeding ecology and a distinguishing character of major groups [18,36,37]. Traditionally, lingual prehension was considered the plesiomorphic condition, with jaw prehension evolving in ‘scleroglossans’ or non-iguanian squamates [18]. Morphological and functional differences in the hyolingual apparatus have been identified between lingual and jaw feeders [36], but mandibular morphology has not been quantitatively compared before our study. We find that prehension mechanism does not have a significant impact on variation in lower jaw morphology among lepidosaurs, and that lingual-feeding and jaw-feeding lizards show similar levels of mandibular disparity and evolutionary rates. This suggests that mandibular shape is not important in the type of prey capture, and that adaptations for lingual and jaw prehension might lie in other aspects of the feeding apparatus, such as the hyoid apparatus. In contrast with the traditional hypothesis, our ancestral state reconstruction of prehension modes supports that the ancestral condition for both Lepidosauria and Squamata is jaw prehension, and that lingual prehension is an evolutionary innovation of Iguania. This implies that lingual prehension has evolved independently in Iguania and *Sphenodon* [85], and unfortunately it is impossible to know at which stage this character evolved during the evolution of Rhynchocephalia. Lingual prehension in Iguania has been considered an evolutionarily stable configuration or a combination of characters that perform a certain function and remain unchanged over long macroevolutionary scales [38]. Instead of lingual prehension being a plesiomorphic condition stabilized in iguanians, our findings suggest that this suite of characters evolved at the origin of the clade from an ancestral jaw prehension state. Additionally, the high probability for both types of prehension being ancestral to Scincoidea agrees with an increasing number of studies describing mixed capture strategies in more species within this clade [43–45].

The tempo of morphological evolution of the mandible is variable across the phylogeny of Lepidosauria. High evolutionary rates are mostly concentrated within Iguania and Serpentes, and major clades that show generalized low rates of evolution, such as Gekkota and Scincoidea, include certain lineages with peculiar skull morphologies that evolved at faster rates. For instance, increased evolutionary rates are seen in the branch leading to the limbless pygopodid gecko *Lialis*, which has evolved extremely elongated jaws that allow a unique feeding behaviour among geckoes, consisting of capturing and consuming large prey [86]. In snakes, high evolutionary rates of the mandibles occur in nested clades of viperids and colubrids, while rapid evolution of the cranium mostly concentrates at the origin of the clade [33], suggesting that substantial modifications of the mandible occurred at later stages in snake evolution compared with the cranium. High rates of jaw evolution are found in the sea snake *Hydrophis platurus*, characterized by its long and narrow skull, and in the diverse lineage of colubrid snakes, particularly in the genus *Pituophis*. Vipers also show sustained fast rates of evolution responsible for the characteristic elongation of their lower jaws compared with other snakes, especially in the genus *Crotalus*, a feature that contributes to their ability to swallow large prey relative to their body size [87]. Within Iguania, acrodontans show generalized low rates of evolution except for the bearded dragon, *Pogona*. The low to medium evolutionary rates of jaw shape seen in chameleons contrast with fast evolutionary rates in their crania [33], suggesting fast evolution promoted the acquisition of their highly specialized cranial morphology but nor their relatively generalized mandibular shape. Finally, two groups of pleurodontans show a burst of jaw shape evolution: the phrynosomatids and liolaemids. The genus *Phrynosoma* is a diverse clade that contains ant-eating specialists with unique skull morphologies characterized by short and deep skulls with bony horns, large orbits and reduced tooth rows. A short jaw with a small coronoid process, reduced jaw muscles and low bite forces (except for some beetle specialists) have evolved in this group as adaptations to a myrmecophagous diet [88,89]. Our analyses provide evidence not only that phrynosomatids have unique jaw morphologies among lepidosaurs, but that sustained rapid evolutionary modifications of the mandibles have been key to their radiation into specialized ecological niches. Liolaemids, a diverse and recent radiation of South American lizards, show heterogeneous but overall high rates of jaw evolution. Members of this clade have adapted to diverse environments and ecologies, including different kinds of dietary habits, through remarkable innovation in body shape [66,90]. The rapid evolution of mandibular shape in most species of *Liolaemus* is likely related to this exploration of dietary habits that allowed their conquest of diverse and extreme environments [26]. Overall, the distribution of evolutionary rates of mandibular shape suggests that rapid evolution has promoted ecomorphological specialization or diversification in lepidosaurs.

## 5. Conclusions

Throughout their evolutionary history, lepidosaurs have radiated into an outstanding array of morphologies and ecologies, becoming the most diverse group of modern tetrapods. Our study provides the broadest characterization of the morphological diversity of the mandibular apparatus in the clade, identifying its main evolutionary drivers. Our findings highlight the complex interplay of driving factors behind the evolution of the mandible, a multifunctional structure representing a fundamental component of the feeding apparatus. Despite its expected tight link with ecology, we find that high-level factors such as phylogeny and allometry impose significant constraints on this structure. At the same time, our study shows that evolutionary lability in patterns of jaw shape disparity and evolutionary rates contributes to ecological diversification, suggesting that morphological innovation in the mandible plays a key role in the ecomorphological radiation of extremely diverse clades.

## Data Availability

Data (code, analyses files and CT scan data of specimens scanned in this study) are available at the University of Bristol data repository, data.bris, at [91]. Supplementary material is available online [92].

## References

[1] Lewontin RC . 1978 Adaptation. Scient. Am. **239** , 212–218, (10.1038/scientificamerican0978-212) 705323

[2] Seilacher A . 1970 Arbeitskonzept zur Konstruktions‐Morphologie. Lethaia **3** , 393–396. [In German, with an English abstract]. (10.1111/j.1502-3931.1970.tb00830.x)

[3] Gould SJ , Lewontin RC . 1979 The spandrels of San Marco and the Panglossian paradigm: a critique of the adaptationist programme. Proc. R. Soc. B **205** , 581–598. (10.1098/rspb.1979.0086) 42062

[4] Alberch P . 1980 Ontogenesis and morphological diversification. Am. Zool. **20** , 653–667. (10.1093/icb/20.4.653)

[5] Losos JB . 2008 Phylogenetic niche conservatism, phylogenetic signal and the relationship between phylogenetic relatedness and ecological similarity among species. Ecol. Lett. **11** , 995–1003. (10.1111/j.1461-0248.2008.01229.x) 18673385

[6] Pavón-Vázquez CJ , Esquerré D , Keogh JS . 2022 Ontogenetic drivers of morphological evolution in monitor lizards and allies (Squamata: Paleoanguimorpha), a clade with extreme body size disparity. BMC Ecol. Evol. **22** , 15. (10.1186/s12862-022-01970-6) 35151266 PMC8840268

[7] Klaczko J , Sherratt E , Setz EZF . 2016 Are diet preferences associated to skulls shape diversification in xenodontine snakes? PLoS One **11** , e0148375. (10.1371/journal.pone.0148375) 26886549 PMC4757418

[8] Uetz P , Freed P , Aguilar R , Reyes F , Kudera J , Hošek J . 2023 The reptile database. See http://www.reptile-database.org.

[9] Jones MEH , Anderson CL , Hipsley CA , Müller J , Evans SE , Schoch RR . 2013 Integration of molecules and new fossils supports a Triassic origin for Lepidosauria (lizards, snakes, and tuatara). BMC Evol. Biol. **13** , 208. (10.1186/1471-2148-13-208) 24063680 PMC4016551

[10] Feldman A , Sabath N , Pyron RA , Mayrose I , Meiri S . 2016 Body sizes and diversification rates of lizards, snakes, amphisbaenians and the tuatara. Glob. Ecol. Biogeogr. **25** , 187–197. (10.1111/geb.12398)

[11] Polcyn MJ , Jacobs LL , Araújo R , Schulp AS , Mateus O . 2014 Physical drivers of mosasaur evolution. Palaeogeogr. Palaeoclimatol. Palaeoecol. **400** , 17–27. (10.1016/j.palaeo.2013.05.018)

[12] Wiens JJ , Brandley MC , Reeder TW . 2006 Why does a trait evolve multiple times within a clade? Repeated evolution of snakelike body form in squamate reptiles. Evolution **60** , 123–141. (10.1111/j.0014-3820.2006.tb01088.x) 16568638

[13] Cundall D , Irish F . 2008 The snake skull. In Biology of the Reptilia: volume 20, Morphology H: The skull of Lepidosauria (eds C Gans , AS Gaunt , K Adler ), pp. 349–692. Ithaca, NY: Society for the Study of Amphibians and Reptiles.

[14] Evans SE . 2008 The skull of lizards and tuatara. In Biology of the Reptilia: volume 20, Morphology H: The skull of Lepidosauria (eds C Gans , AS Gaunt , K Adler ), pp. 1–348. Ithaca, NY: Society for the Study of Amphibians and Reptiles.

[15] Rieppel O , Gronowski RW . 1981 The loss of the lower temporal arcade in diapsid reptiles. Zool. J. Linn. Soc. **72** , 203–217. (10.1111/j.1096-3642.1981.tb01570.x)

[16] Herrel A , Schaerlaeken V , Meyers JJ , Metzger KA , Ross CF . 2007 The evolution of cranial design and performance in squamates: consequences of skull-bone reduction on feeding behavior. Integr. Comp. Biol. **47** , 107–117. (10.1093/icb/icm014) 21672824

[17] Metzger K . 2002 Cranial kinesis in lepidosaurs: skulls in motion. In Topics in functional and ecological vertebrate morphology (eds P Aerts , K D’Août , A Herrel , R Van Damme ), pp. 15–46. Maastricht, The Netherlands: Shaker.

[18] Vitt LJ , Pianka ER , Cooper Jr WE , Schwenk K . 2003 History and the global ecology of squamate reptiles. Am. Nat. **162** , 44–60. (10.1086/375172) 12856236

[19] Pyron RA . 2014 Temperate extinction in squamate reptiles and the roots of latitudinal diversity gradients. Glob. Ecol. Biogeogr. **23** , 1126–1134. (10.1111/geb.12196)

[20] Tulli MJ , Abdala V , Cruz FB . 2011 Relationships among morphology, clinging performance and habitat use in liolaemini lizards. J. Evol. Biol. **24** , 843–855. (10.1111/j.1420-9101.2010.02218.x) 21255177

[21] Kulyomina Y , Moen DS , Irschick DJ . 2019 The relationship between habitat use and body shape in geckos. J. Morphol. **280** , 722–730. (10.1002/jmor.20979) 30950546

[22] Morinaga G , Bergmann PJ . 2020 Evolution of fossorial locomotion in the transition from tetrapod to snake-like in lizards. Proc. R. Soc. B **287** , 20200192. (10.1098/rspb.2020.0192) PMC7126036 32183623

[23] Bauer AM , Jackman T . 2008 Global diversity of lizards in freshwater (Reptilia: Lacertilia). Hydrobiologia **595** , 581–586. (10.1007/s10750-007-9115-0)

[24] Murphy JC . 2012 Marine invasions by non-sea snakes, with thoughts on terrestrial–aquatic–marine transitions. Integr. Comp. Biol. **52** , 217–226. (10.1093/icb/ics060) 22576813

[25] Grundler MC , Rabosky DL . 2021 Rapid increase in snake dietary diversity and complexity following the end-Cretaceous mass extinction. PLoS Biol. **19** , e3001414. (10.1371/journal.pbio.3001414) 34648487 PMC8516226

[26] Ocampo M , Pincheira-Donoso D , Sayol F , Rios RS . 2022 Evolutionary transitions in diet influence the exceptional diversification of a lizard adaptive radiation. BMC Ecol. Evol. **22** , 74. (10.1186/s12862-022-02028-3) 35672668 PMC9175459

[27] Cooper WE , Vitt LJ . 2002 Distribution, extent, and evolution of plant consumption by lizards. J. Zool. **257** , 487–517. (10.1017/S0952836902001085)

[28] Espinoza RE , Wiens JJ , Tracy CR . 2004 Recurrent evolution of herbivory in small, cold-climate lizards: breaking the ecophysiological rules of reptilian herbivory. Proc. Natl Acad. Sci. USA **101** , 16819–16824. (10.1073/pnas.0401226101) 15550549 PMC534712

[29] Hanken J , Hall BK . 1993 The skull, volume 3: Functional and evolutionary mechanisms. Chicago, IL: University of Chicago Press.

[30] Stayton CT . 2005 Morphological evolution of the lizard skull: a geometric morphometrics survey. J. Morphol. **263** , 47–59. (10.1002/jmor.10288) 15536647

[31] Stayton CT . 2006 Testing hypotheses of convergence with multivariate data: morphological and functional convergence among herbivorous lizards. Evolution **60** , 824–841. (10.1111/j.0014-3820.2006.tb01160.x) 16739463

[32] Da Silva FO , Fabre AC , Savriama Y , Ollonen J , Mahlow K , Herrel A , Müller J , Di-Poï N . 2018 The ecological origins of snakes as revealed by skull evolution. Nat. Commun. **9** , 376. (10.1038/s41467-017-02788-3) 29371624 PMC5785544

[33] Watanabe A , Fabre AC , Felice RN , Maisano JA , Müller J , Herrel A , Goswami A . 2019 Ecomorphological diversification in squamates from conserved pattern of cranial integration. Proc. Natl Acad. Sci. USA **116** , 14688–14697. (10.1073/pnas.1820967116) 31262818 PMC6642379

[34] Ollonen J *et al* . 2024 Dynamic evolutionary interplay between ontogenetic skull patterning and whole-head integration. Nat. Ecol. Evol. **8** , 536–551. (10.1038/s41559-023-02295-3) 38200368

[35] Maynard Smith J , Savage RJG . 1959 The mechanics of mammalian jaws. School Sci. Rev. **141** , 289–301.

[36] Schwenk K , Throckmorton GS . 1989 Functional and evolutionary morphology of lingual feeding in squamate reptiles: phylogenetics and kinematics. J. Zool. **219** , 153–175. (10.1111/j.1469-7998.1989.tb02573.x)

[37] Schwenk K . 2000 Feeding in lepidosaurs. In Feeding: form, function and evolution in tetrapod vertebrates (ed. K Schwenk ), pp. 175–291. San Diego, CA: Academic Press.

[38] Schwenk K , Wagner GP . 2001 Function and the evolution of phenotypic stability: connecting pattern to process. Am. Zool. **41** , 552–563. (10.1093/icb/41.3.552)

[39] Gorniak GC , Rosenberg HI , Gans C . 1982 Mastication in the tuatara, Sphenodon punctatus (Reptilia: Rhynchocephalia): structure and activity of the motor system. J. Morphol. **171** , 321–353. (10.1002/jmor.1051710307) 30089350

[40] Pyron RA , Burbrink FT , Wiens JJ . 2013 A phylogeny and revised classification of Squamata, including 4161 species of lizards and snakes. BMC Evol. Biol. **13** , 93. (10.1186/1471-2148-13-93) 23627680 PMC3682911

[41] Tonini JFR , Beard KH , Ferreira RB , Jetz W , Pyron RA . 2016 Fully-sampled phylogenies of squamates reveal evolutionary patterns in threat status. Biol. Conserv. **204** , 23–31. (10.1016/j.biocon.2016.03.039)

[42] Title PO *et al* . 2024 The macroevolutionary singularity of snakes. Science **383** , 918–923. (10.1126/science.adh2449) 38386744

[43] Urbani J‐M , Bels VL . 1995 Feeding behaviour in two scleroglossan lizards: Lacerta viridis (Lacertidae) and Zonosaurus laticaudatus (Cordylidae). J. Zool. **236** , 265–290. (10.1111/j.1469-7998.1995.tb04493.x)

[44] Broeckhoven C , Mouton P le FN . 2013 Influence of diet on prehension mode in cordylid lizards: a morphological and kinematic analysis. J. Zool. **291** , 286–295. (10.1111/jzo.12075)

[45] Hewes AE , Schwenk K . 2021 The functional morphology of lingual prey capture in a scincid lizard, Tiliqua scincoides (Reptilia: Squamata). J. Morphol. **282** , 127–145. (10.1002/jmor.21287) 33090536

[46] Fabre AC , Dowling C , Portela Miguez R , Fernandez V , Noirault E , Goswami A . 2021 Functional constraints during development limit jaw shape evolution in marsupials. Proc. R. Soc. B **288** , 20210319. (10.1098/rspb.2021.0319) PMC8079998 33906406

[47] López-Romero FA , Stumpf S , Kamminga P , Böhmer C , Pradel A , Brazeau MD , Kriwet J . 2023 Shark mandible evolution reveals patterns of trophic and habitat-mediated diversification. Commun. Biol. **6** , 496. (10.1038/s42003-023-04882-3) 37156994 PMC10167336

[48] Cardini A . 2017 Left, right or both? Estimating and improving accuracy of one-side-only geometric morphometric analyses of cranial variation. J. Zool. Syst. Evol. Res. **55** , 1–10. (10.1111/jzs.12144)

[49] Botton-Divet L , Cornette R , Fabre AC , Herrel A , Houssaye A . 2016 Morphological analysis of long bones in semi-aquatic mustelids and their terrestrial relatives. Integr. Comp. Biol. **56** , 1298–1309. (10.1093/icb/icw124) 27794537

[50] Gunz P , Mitteroecker P , Bookstein FL . 2005 Semilandmarks in three dimensions. In Modern morphometrics in physical anthropology (ed. DE Slice ), pp. 73–98. Boston, MA: Springer. (10.1007/0-387-27614-9_3)

[51] Schlager S , Jefferis G , Ian D . 2013 Morpho: calculations and visualisations related to geometric morphometrics. R package v 29. See https://CRAN.R-project.org/package=Morpho.

[52] Adams DC , Otárola‐Castillo E . 2013 geomorph: An R package for the collection and analysis of geometric morphometric shape data . Methods Ecol. Evol. **4** , 393–399. (10.1111/2041-210X.12035)

[53] Revell LJ . 2009 Size-correction and principal components for interspecific comparative studies. Evolution **63** , 3258–3268. (10.1111/j.1558-5646.2009.00804.x) 19663993

[54] Adams DC . 2014 A generalized K statistic for estimating phylogenetic signal from shape and other high-dimensional multivariate data. Syst. Biol. **63** , 685–697. (10.1093/sysbio/syu030) 24789073

[55] Revell LJ . 2012 phytools: An R package for phylogenetic comparative biology (and other things). Methods Ecol. Evol. **3** , 217–223. (10.1111/j.2041-210X.2011.00169.x)

[56] Milla Carmona P . 2023 morphospace: Build, visualize and explore multivariate ordinations of shape data. See https://github.com/millacarmona/morphospace.

[57] Castiglione S , Tesone G , Piccolo M , Melchionna M , Mondanaro A , Serio C , Di Febbraro M , Raia P . 2018 A new method for testing evolutionary rate variation and shifts in phenotypic evolution. Methods Ecol. Evol. **9** , 974–983. (10.1111/2041-210X.12954)

[58] Castiglione S *et al* . 2019 A new, fast method to search for morphological convergence with shape data. PLoS One **14** , e0226949. (10.1371/journal.pone.0226949) 31881075 PMC6934287

[59] Guillerme T . 2018 dispRity: a modular R package for measuring disparity. Methods Ecol. Evol. **9** , 1755–1763. (10.1111/2041-210X.13022)

[60] Louca S , Doebeli M . 2018 Efficient comparative phylogenetics on large trees. Bioinformatics **34** , 1053–1055. (10.1093/bioinformatics/btx701) 29091997

[61] Clavel J , Escarguel G , Merceron G . 2015 mvMORPH: an R package for fitting multivariate evolutionary models to morphometric data. Methods Ecol. Evol. **6** , 1311–1319. (10.1111/2041-210X.12420)

[62] Gelman A , Rubin DB . 1992 Inference from iterative simulation using multiple sequences. Stat. Sci. **7** , 457–472. (10.1214/ss/1177011136)

[63] Plummer M , Best N , Cowles K , Vines K . 2006 CODA: convergence diagnosis and output analysis for MCMC. R News **6** , 7–11.

[64] Ricklefs RE , Miles DB . 1994 Ecological and evolutionary inferences from morphology: an ecological perspective. In Ecological morphology: integrative organismal biology (eds PC Wainwright , SM Reilly ), pp. 13–41. Chicago, IL: University of Chicago Press.

[65] Lauder GV . On the inference of function from structure. In Functional morphology in vertebrate paleontology (ed. J Thomason ), pp. 1–18. Cambridge, UK: Cambridge University Press.

[66] Harmon LJ , Schulte JA , Larson A , Losos JB . 2003 Tempo and mode of evolutionary radiation in iguanian lizards. Science **301** , 961–964. (10.1126/science.1084786) 12920297

[67] Cundall D , Greene HW . 2000 Feeding in snakes. In Feeding: form, function, and evolution in tetrapod vertebrates (ed. K Schwenk ), pp. 293–333. San Diego, CA: Academic Press.

[68] Rodríguez‐Robles JA , Bell CJ , Greene HW . 1999 Gape size and evolution of diet in snakes: feeding ecology of erycine boas. J. Zool. **248** , 49–58. (10.1111/j.1469-7998.1999.tb01021.x)

[69] Cundall D . 1995 Feeding behaviour in Cylindrophis and its bearing on the evolution of alethinophidian snakes. J. Zool. **237** , 353–376. (10.1111/j.1469-7998.1995.tb02767.x)

[70] Gray JA , Sherratt E , Hutchinson MN , Jones MEH . 2019 Evolution of cranial shape in a continental-scale evolutionary radiation of Australian lizards. Evolution **73** , 2216–2229. (10.1111/evo.13851) 31580481

[71] Hipsley CA , Müller J . 2017 Developmental dynamics of ecomorphological convergence in a transcontinental lizard radiation. Evolution **71** , 936–948. (10.1111/evo.13186) 28085191

[72] Carrasco PA , Prystupczuk L , Koch C , González GA , Leynaud GC , Grazziotin FG . 2023 Patterns of morphological variation and ecological correlates in the skull of vipers (Serpentes: Viperidae). J. Morphol. **284** , e21617. (10.1002/jmor.21617) 37458083

[73] Gans C . 1961 The feeding mechanism of snakes and its possible evolution. Am. Zool. **1** , 217–227. (10.1093/icb/1.2.217)

[74] Santana SE , Grosse IR , Dumont ER . 2012 Dietary hardness, loading behavior, and the evolution of skull form in bats. Evolution **66** , 2587–2598. (10.1111/j.1558-5646.2012.01615.x) 22834755

[75] Dumont M , Wall CE , Botton-Divet L , Goswami A , Peigné S , Fabre AC . 2016 Do functional demands associated with locomotor habitat, diet, and activity pattern drive skull shape evolution in musteloid carnivorans? Biol. J. Linn. Soc. **117** , 858–878. (10.1111/bij.12719)

[76] Meloro C , Cáceres NC , Carotenuto F , Sponchiado J , Melo GL , Passaro F , Raia P . 2015 Chewing on the trees: constraints and adaptation in the evolution of the primate mandible. Evolution **69** , 1690–1700. (10.1111/evo.12694) 26095445

[77] Bright JA , Marugán-Lobón J , Rayfield EJ , Cobb SN . 2019 The multifactorial nature of beak and skull shape evolution in parrots and cockatoos (Psittaciformes). BMC Evol. Biol. **19** , 104. (10.1186/s12862-019-1432-1) 31101003 PMC6525378

[78] Metzger KA , Herrel A . 2005 Correlations between lizard cranial shape and diet: a quantitative, phylogenetically informed analysis. Biol. J. Linn. Soc. **86** , 433–466. (10.1111/j.1095-8312.2005.00546.x)

[79] Herrel A , Aerts P De Vree F 1998 Ecomorphology of the lizard feeding apparatus: a modelling approach. Neth. J. Zool. **48** , 1–25.

[80] Saulnier Masson R , Daoues K , Measey J , Herrel A . 2023 The evolution of bite force and head morphology in scincid lizards: diet and habitat use as possible drivers. Biol. J. Linn. Soc. **140** , 58–73. (10.1093/biolinnean/blad052)

[81] Lafuma F , Corfe IJ , Clavel J , Di-Poï N . 2021 Multiple evolutionary origins and losses of tooth complexity in squamates. Nat. Commun. **12** , 6001. (10.1038/s41467-021-26285-w) 34650041 PMC8516937

[82] Barros FC , Herrel A , Kohlsdorf T . 2011 Head shape evolution in Gymnophthalmidae: does habitat use constrain the evolution of cranial design in fossorial lizards? J. Evol. Biol. **24** , 2423–2433. (10.1111/j.1420-9101.2011.02372.x) 21883615

[83] Segall M , Cornette R , Fabre AC , Godoy-Diana R , Herrel A . 2016 Does aquatic foraging impact head shape evolution in snakes? Proc. R. Soc. B **283** , 20161645. (10.1098/rspb.2016.1645) PMC5013807 27581887

[84] Anelli V , Bars‐Closel M , Herrel A , Kohlsdorf T . 2024 Different selection regimes explain morphological evolution in fossorial lizards. Funct. Ecol. **38** , 1250–1264. (10.1111/1365-2435.14557)

[85] Vidal N , Hedges SB . 2009 The molecular evolutionary tree of lizards, snakes, and amphisbaenians. C. R. Biol. **332** , 129–139. (10.1016/j.crvi.2008.07.010) 19281946

[86] Patchell FC , Shine R . 1986 Feeding mechanisms in pygopodid lizards: how can Lialis swallow such large prey? J. Herpetol. **20** , 59–64. (10.2307/1564125)

[87] Pough FH , Groves JD . 1983 Specializations of the body form and food habits of snakes. Am. Zool. **23** , 443–454. (10.1093/icb/23.2.443)

[88] Montanucci RR . 1989 The relationship of morphology to diet in the horned lizard genus Phrynosoma. Herpetologica **45** , 208–216.

[89] Meyers JJ , Nishikawa KC , Herrel A . 2018 The evolution of bite force in horned lizards: the influence of dietary specialization. J. Anat. **232** , 214–226. (10.1111/joa.12746) 29159806 PMC5770303

[90] Edwards DL , Avila LJ , Martinez L , Sites Jr JW , Morando M . 2022 Environmental correlates of phenotypic evolution in ecologically diverse Liolaemus lizards. Ecol. Evol. **12** , e9009. (10.1002/ece3.9009) 35784059 PMC9201750

[91] Ballell A , Dutel H , Fabbri M , Martin-Silverstone E , Kersley A , Hammond CL , Herrel A , Rayfield EJ . 2024 Data from: Ecological drivers of jaw morphological evolution in lepidosaurs. Data.bris. (10.5523/bris.2t3lur8am5chu2ovswdb92qs5d) PMC11641439 39657804

[92] Ballell A , Dutel H , Fabbri M , Martin-Silverstone E , Kersley A , Hammond C . 2024 Supplementary material from: Ecological drivers of jaw morphological evolution in lepidosaurs. Figshare. (10.6084/m9.figshare.c.7565516) PMC11641439 39657804

